# Role of Regulatory Immune Cells and Molecules in Autoimmune Bullous Dermatoses

**DOI:** 10.3389/fimmu.2019.01746

**Published:** 2019-08-02

**Authors:** Tianyu Cao, Shuai Shao, Hui Fang, Bing Li, Gang Wang

**Affiliations:** ^1^Department of Dermatology, Tangdu Hospital, Fourth Military Medical University, Xi'an, China; ^2^Department of Dermatology, Xijing Hospital, Fourth Military Medical University, Xi'an, China

**Keywords:** regulatory T cells, regulatory B cells, complement regulating proteins, pemphigus vulgaris, bullous pemphigoid

## Abstract

Autoimmune bullous dermatoses (AIBD) include a series of typical organ-specific autoimmune diseases characterized by extensive mucocutaneous blisters. It is generally accepted to be caused by pathological autoantibodies that directly target specific adhesion components of the skin or the adjacent mucous membranes. Both innate and adaptive immune systems are critically involved in the misguided immune response against self-antigens. Recent studies have indicated that the dysfunction of regulatory T cells, regulatory B cells, and complement regulatory proteins that play essential roles in maintaining a healthy immune environment is also closely related to immune disorders in AIBD. It is important to summarize these studies, elucidate the changes in these regulatory immune cells and molecules for the pathogenesis of AIBD, and reveal the mechanisms by which they lose their ability to regulate immune disorders. In this review, we highlight the role of regulatory immune cells and molecules in the pathogenesis of pemphigus vulgaris and bullous pemphigoid, the two most representative forms of AIBD, and indicate issues that should be addressed in future investigations.

## Introduction

The immune system is guided by a series of regulatory mechanisms, the main components of which are a large number of immunoreactive and suppressor cells, that modulate the host response (1). Various immune cells and molecules that have been found to possess a regulatory capacity include regulatory T (Treg) cells, regulatory B (Breg) cells, macrophages, myeloid-derived suppressor cells (MDSCs), dendritic cells (DCs), mesenchymal stromal cells (MSCs), and complement regulatory proteins (CRPs) (2). Alteration or disruption of these immune regulatory mechanisms may lead to the survival and activation of autoreactive lymphocytes upon encountering appropriate self-antigens, which may cause corresponding tissue and organ damage (2). For instance, regulatory immune mechanisms have been implicated in the development of many autoimmune disorders, such as systemic lupus erythematosus (SLE), rheumatoid arthritis, and multiple sclerosis (3–5).

Autoimmune bullous dermatoses (AIBD) include a series of diseases that cause blistering of the skin and mucous membranes through autoantibody attacks on keratinocyte junction structures or on components of the dermal-epidermal junction (6). Based on the location of its formation, AIBD can be divided into two subgroups: pemphigus and pemphigoid diseases (7). Pemphigus diseases are characterized by the production of IgG autoantibodies against keratinocyte adhesion molecules, resulting in intraepidermal blistering, flaccid vesicles, and erosions of the skin and/or mucous membranes. Diseases in this group include pemphigus vulgaris (PV), pemphigus foliaceus, paraneoplastic pemphigus, and IgA pemphigus (6). In contrast, pemphigoid diseases are characterized by autoantibodies that directly attack structural proteins within the dermal-epidermal junction, leading to urticarial lesions, and tense blisters on the skin (8). This group includes bullous pemphigoid (BP), linear IgA bullous dermatosis, dermatitis herpetiformis, mucous membrane pemphigoids, herpes gestationis, and epidermolysis bullosa acquisita (7).

Recent studies have shown several regulatory immune cells and molecules to be involved in the progression of AIBD. In this review, we aim to elucidate the different mechanisms and roles of regulatory immune cells and molecules in AIBD by summarizing and discussing the findings of various clinical and experimental studies, and outline some of the challenges that lie ahead.

## Clinical Presentation and Pathophysiology of AIBD

### Clinical and Immunohistological Features

**Figure 1:**
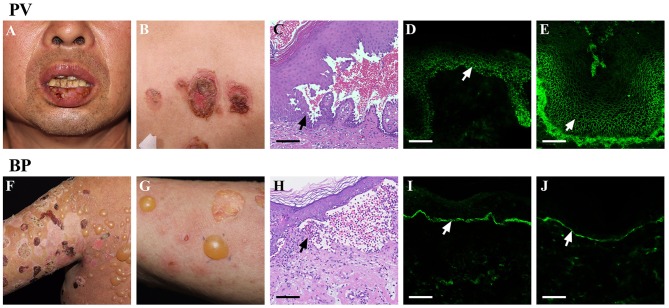
Clinical and immunohistological features of AIBD. AIBD contains two groups: pemphigus group and pemphigoid group. The representative members of these two groups are PV and BP. PV can present with erosions on the mucous membranes **(A)**, and flaccid vesicles or erosion on the skin **(B)**. Haematoxylin and eosin (HandE) staining of PV lesions demonstrates an intraepithelial blister with acantholysis **(C)**. DIF of PV lesions shows IgG antibodies deposit on the surface of keratinocytes **(D)**. IIF using monkey esophagus shows serum IgG autoantibodies from patients with PV bind to the surface of epitheliums **(E)**. Whereas, BP presents with large, tense bullae, and erythematous patches on the skin **(F,G)**. H & E staining of BP lesions reveals an subepidermal blister with eosinophil-rich inflammatory infiltration in the superficial dermis **(H)**. DIF of BP lesions shows IgG antibodies deposit along the dermoepidermal junction **(I)**. IIF using normal skin shows the presence of serum IgG antibodies form BP patients bound to the dermoepidermal junction **(J)**. Bar = 100 μm.

The pemphigus group of AIBD contains several rare organ specific autoimmune diseases that affect the skin and mucous membranes (9). PV, as the most common representative disease of the pemphigus group (10), is characterized by extremely painful erosions of the mucous membranes, and flaccid blisters localized mainly in the flexural areas, face, scalp, and extremities (Figures 1A,B). Typical lesional biopsy specimens of PV show acantholysis, which can progress to the formation of non-inflammatory intraepithelial blisters; however, a few eosinophils or neutrophils can be occasionally seen in the upper dermis and epidermis as well (Figure 1C). Meanwhile, direct immunofluorescence (DIF) of perilesional biopsy specimens from patients with PV shows IgG antibodies, and infrequently complement C3 protein, deposits on the surface of keratinocytes (Figure 1D). Whereas, DIF of normal skin shows no specific fluorescence (Supplementary Figure 1). Indirect immunofluorescence (IIF) using monkey or human normal skin as substrates enables a semiquantitative detection of circulating autoantibodies in serum. And IIF typically shows serum IgG autoantibodies from patients with PV bound to the surface of epithelium (Figure 1E).

BP is the most frequently occurring representative of the pemphigoid group, and is clinically characterized by tense, serous, or hemorrhagic bullae on erythematous or apparently normal skin (Figures 1F,G). The lesional biopsy specimens of BP typically reveal sub-epidermal blister formation with eosinophil and/or neutrophil-rich inflammatory infiltration in the superficial dermis (Figure 1H). In addition, DIF of BP lesions shows the deposits of IgG and/or C3 along the basement membrane zone (BMZ) (Figure 1I), and IgA and IgE were occasionally seen in a similar pattern (11). IIF also shows circulating IgG antibodies form patients with BP bound to the dermoepidermal junction (Figure 1J).

### Genetic Factors

Although the genetic background of AIBD has not been precisely determined, a genetic predisposition in the etiology is evident. The human HLA class I and II molecules play a critical role in the recognition of antigenic peptides by T cells. Several HLA alleles, including HLA-DRB1^*^0402 (in Jewish people) and HLA-DQB1^*^0503 (in non-Jewish populations), have been found to be strongly associated with PV (12). Meanwhile, in BP, HLA-DQB1^*^03:01 is considered the most common HLA class II allele, associated with patients in multiple populations (13–15). Other HLA alleles, such as HLADRB1^*^04, DRB1^*^1101, and DQB1^*^0302, were more frequent in Japanese patients with BP (16), suggesting different HLA class II haplotypes to genetically influence susceptibility to BP across different populations.

### Autoantibody Mediated Blister Formation

The binding of autoantibodies to antigens is universally known as the main cause of blister formation in AIBD. The autoantibodies in PV are against the members of cadherin family, desmoglein (Dsg) 1, and Dsg3, which maintain intercellular adhesion in stratified squamous epithelia (17, 18). The main mechanism of blister formation in PV is steric hindrance, since atomic force microscopy experiments had shown that PV IgG can directly inhibit Dsg3-mediated transinteraction (19). Besides this, several cell signaling pathways and factors are also involved in the blister formation in PV, including p38 mitogen-activated protein kinase (MAPK) (20), c-Myc (21), epidermal growth factor receptor (22), caspases (23), and mitochondria (24).

The autoantibodies in BP target two components of the hemidesmosome adhesion complex, BP180 (collagen XVII) and BP230 (dystonin-e), and the non-collagenous 16A (NC16A) domain of BP180 contains the major epitopes recognized by autoreactive T and B cells (25). In contrast to that in PV, the formation of sub-epidermal blisters in BP depends mainly on the autoantibody-induced inflammation, including activation of complement, degranulation of mast cells (MCs), and activation of neutrophils, since the knockdown of C4 (component of classical complement activation pathway), C5aR (expression on MCs), MC protease-4 (homolog of the human MC chymase), and FcγRIII (the main receptor on neutrophils) were, respectively, resistant to anti-BP180 antibody induced blistering in mice (26–30). Besides, neutrophil elastase, matrix metalloproteinase-9, and other proteolytic enzymes released by neutrophils could directly split the epidermis from the dermis (31).

### Animal Models

Although no single animal model could completely simulate the entire process of PV and BP, several models have been developed for different areas of research. For PV, passive transfer with circulating IgG antibodies, from patients with PV, could induce a PV-like phenotype in neonatal mice (32), which can be used to study the function of autoantibodies. In addition, adoptive transfer of peripheral lymphocytes from Dsg3^−/−^ mice to Rag-2^−/−^ immunodeficient mice could also develop a PV-like phenotype (33). This model may be used to isolate anti-Dsg3 monoclonal antibodies, investigate the roles of T and B lymphocytes in perpetuating autoantibody production for autoimmune response, and evaluate immunosuppressive therapeutic strategies (34, 35).

On the other hand, passive transfer with IgG autoantibodies from patients with BP could not induce a BP-like disease in animals, since BP autoantibodies reacting with NC16A domain failed to cross-react with mouse BP180. In 1993, Liu et al. had reported an experimental BP model by transferring rabbit anti-mouse BP180 NC14A into neonatal mice, which developed the same clinical, histological, and immunopathological features of BP (36). This model has been widely used to investigate the roles of autoantibodies, complement, MCs, neutrophils, and FcγRs in BP (28, 30, 37). Additionally, in humanized mouse whose murine BP180NC14A is replaced with the homologous human BP180NC16A epitope cluster region, injection of anti-BP180NC16A autoantibodies result in the development of BP-like subepidermal blisters (25).

## Treg Cells in AIBD

### CD4^+^ CD25^Bright^ FoxP3^+^ Natural Treg (nTreg) Cells

The pathogenetic progress of AIBD includes a series of immune events, including activation of autoreactive T cells, T–B cell interaction, autoantibody production, and blister formation (38). Recent studies have indicated the involvement of regulatory immune cells and molecules as well in AIBD. Although a wealth of regulatory immune cells have been identified, CD4^+^ CD25^bright^ FoxP3^+^ natural Treg (nTreg) cells remain the most extensively studied cell-type (39). In 1995, Sakaguchi et al. firstly reported and defined a subset of CD4^+^ cells showing stable expression of the lineage-specific transcription factor FoxP3 and high concentrations of the interleukin 2 (IL2) receptor α-chain (CD25) (40). CD4^+^ CD25^bright^ FoxP3^+^ Treg cells account for 10% of peripheral CD4^+^ T cells and demonstrate the suppressing function, both in cell contact-dependent and -independent manner (41). CD4^+^ CD25^bright^ FoxP3^+^ Treg cells expressed cytotoxic T-lymphocyte associated protein 4 (CTLA-4), CD25, CD39, and CD73 ectoenzymes, which directly suppressed the production of CD8^+^ T cells, DCs, myeloid cells, and possibly natural killer (NK) cells (42–44). Moreover, CD4^+^ CD25^bright^ FoxP3^+^ Treg cells can also produce a variety of immunomodulatory cytokines, such as IL-10, granzymes, and transforming growth factor-β (TGF-β), which suppress the proinflammatory responses of effector T cells, NK cells, B cells, DCs, and macrophages (45).

#### nTreg Cells in Pemphigus Vulgaris

The concentration and function of CD4^+^ CD25^bright^ FoxP3^+^ Treg cells in patients with PV are abnormal. Sugiyama et al. (46) found that CD4^+^ Treg cells were remarkably reduced in the blood of patients with PV (46). In addition, CD4^+^ CD25 ^bright^ Treg cells in such patients showed significantly higher CD45RO and lower CD45RA expression levels than in normal controls. Moreover, the expression of FoxP3 and CTLA-4 in CD4^+^ CD25 ^bright^ Treg cells was significantly down-regulated in patients with PV compared to that in healthy people, along with dysregulation of CCR4 and CCL22 expression (46, 47), thus suggesting the possibility of defective trafficking of Treg cells in skin lesions of patients with PV. Furthermore, the number of CD4^+^ Treg cells was negatively correlated with that of Th17 cells, which is significantly increased in patients with PV (48). This imbalance might be an important factor in the progression of PV. Importantly, recent studies have indicated that increasing the number of CD4^+^ CD25^bright^ FoxP3^+^ Treg cells, by adoptive transfer or use of the super-agonistic anti-CD28 antibody D665, could suppress antibody production in mouse models of PV, whereas depleting them enhanced the autoantibody production in the same model (49, 50). These results suggested that CD4^+^ CD25^bright^ FoxP3^+^ Treg cell dysfunction promotes the progression of PV.

However, there have been several studies that showed inconsistent results for the change of nTreg cells after treatment. Bhattacharjee et al. found no obvious changes in CD4^+^ CD25^+^ FoxP3^+^ T cell counts in patients with PV after monotherapy with rituximab, an anti-CD20 monoclonal antibody that could induce B cell depletion and is commonly used in autoimmune diseases (51, 52). El-Zawahry et al. however showed that the circulating CD4^+^ CD25^+^ T cells in patients with PV were reduced after treatment with rituximab (53). In several other autoimmune diseases, such as lupus nephritis, idiopathic thrombocytopenic purpura, myasthenia gravis, and rheumatoid arthritis, treatment with rituximab significantly increased the growth of CD4^+^ CD25^bright^ FoxP3^+^ Treg cells (54–57). These discrepancies in the results may be due to the limited number of samples, or differences in the pathogenesis of each disease. Therefore, whether nTreg cells function as a therapy against PV is still debatable.

**Figure 2:**
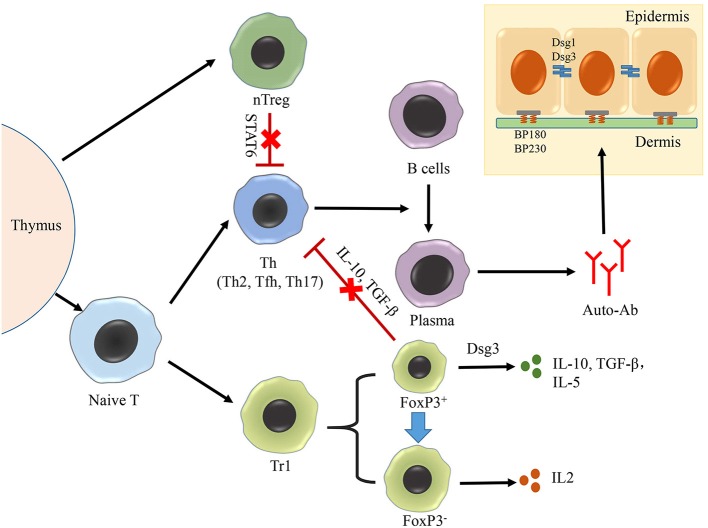
Role of treg cells in AIBD. The production of autoantibodies by plasma cells is the crucial process in the pathogenesis of both PV and BP, in which Th–B cell interaction plays an important role. Treg cells can be divided into two groups: nTreg cells and iTreg cells. nTreg cells number is decreased in PV, which have a negative correlation with Th17 cells. nTreg cells could suppress the functions of Th2 and Tfh cells on activing B cells in an STAT6-dependent manner, but they lost this function in BP. Additionally, Tr1 cells, the most intensively studied cell type of iTreg cells, could be divided into two subpopulations based on the cell size and granularity in PV. The smaller subset achieves the suppressing function by secreting IL-10, TGF-β in response to Dsg3. The larger subset loses the ability in response to Dsg3, exhibits a Th cell-like phenotype, and secretes IL-2.

In brief, defects in the growth and function of nTreg cells, not only reduce the excessive response limit of Th cells, but also promote the production of autoantibodies, thus contributing to the development of PV (Figure 2). However, the molecular mechanism controlling the suppression of nTreg cells, as well as their therapeutic potential, warrants further investigations.

#### nTreg Cells in Bullous Pemphigoid

In patients with BP, the number of CD4^+^ CD25^bright^ FoxP3^+^ Treg cells was found to be significantly reduced, both in the blood and skin (58, 59). In addition, the function of CD4^+^ CD25^bright^ FoxP3^+^ Treg cells in BP has also been found to be abnormal. For instance, a study had shown that scurfy mice, which carry *FoxP3* gene mutations, lacked functional Treg cells, displayed severe erosive skin lesions similar to that in BP, and produced autoantibodies targeting murine BP230 and BP180. Moreover, the transfer of CD4+ T cells from scurfy mice to immunodeficient mice induced the expression of autoantibodies targeting BP230 and BP180, and this phenomenon was ameliorated in *STAT6* gene knockout mice (60). An additional study had discovered that the formation of sub-epidermal blisters in scurfy mice is caused by the monoclonal antibody (mAb) 20B12, which could cross-react with human BP230 (61). These studies together suggest that the absence of FoxP3^+^ Treg cells leads to the BP phenotype in mice by inducing the expression of pathogenic autoantibodies targeting BP antigens (Figure 2). Further research is required to clarify the immunological mechanisms controlling the inhibition of autoantibody production by CD4^+^ Treg cells and to understand why this function is lost in BP.

Another study had shown that CD25^high^ Treg cells were in similar proportions to that of CD4^+^ T cells in BP and healthy blood, and that the ability of CD4^+^ CD25^high^ Treg cells to suppress T cell proliferation and interferon-γ (IFN-γ) secretion in patients with BP was similar to that in normal individuals (62). Although the results seemed to be contradictory, CD4 and CD25, by themselves, are not sufficient to identify Treg cells. CD4^+^ CD25^bright^ cells may represent not only Treg cells, but also FoxP3^−^activated T cells. The inconsistent results may have been caused by the different phenotypes of Treg cells.

### Induced Treg (iTreg) Cells

Besides thymus-derived FoxP3^+^ nTreg cells, there are several other types of Treg cells that can be induced from peripheral naive T lymphocytes in the periphery, including FoxP3^+^ iTreg cells (63), IL-10-producing T regulatory type 1 (Tr1) cells (64), TGF-β-secreting Th3 cells (65), and B-cell-induced Foxp3^−^ regulatory T cells (Treg-of-B cells) (66). Among them, Tr1 cells are the most extensively studied type. Tr1 cells were first reported in 1997 and could produce high levels of IL-10 and TGF-β, which played a key role in suppressing antigen-specific T cell responses (67).

Veldman et al. had shown that Dsg3-responsive Tr1 cells, isolated from healthy carriers of two PV-associated HLA class II alleles (DRB1^*^0402 and DQB1^*^0503), were present in significantly higher proportions than in patients with PV. This population of cells secreted IL-10, TGF-β, and IL-5 in response to Dsg-3, and inhibited the proliferation of Dsg3-reactive Th cells (68). The Dsg3-responsive Tr1 cells could be divided into two subpopulations based on their cell size and granularity. The smaller subset expressed FoxP3 and secreted IL-10 and TGF-β in response to Dsg3 stimulation. The larger subset, on the other hand, did not express FoxP3, exhibited a Th cell-like phenotype, and secreted IL-2 (69) (Figure 2). Inhibition of *FoxP3* mRNA in Tr1, using antisense oligonucleotides resulted in the cells showing features similar to those of Th2 cells, such as the secretion of IL-2 and loss of anergy in response to Dsg3 antigen stimulation (70). After treatment with rituximab, the mean count of Tr1 cells increased in 2 weeks, and gradually declined over the remaining period (51). In summary, alterations in the growth and function of Dsg3- responsive Tr1 cells in PV, as well as their inhibitory effect on Th cells, indicate their relevance in PV pathogenesis. Future studies should focus on the underlying mechanisms controlling phenotypic changes in Tr1 cells during PV pathogenesis, and the development of related PV treatment strategies.

Not enough evidence in AIBD accounts for the changes in other iTreg cell subsets. One study had shown that the proportion of TGF-β-secreting Th3 cells did not change in PV cases compared to that in normal individuals, although there was an increase in the mean value at 14 weeks after treatment with rituximab (51). Since the phenotypic identification on Treg cells has long been inconsistent, reliability of some studies of Treg cells remains debatable. Future studies should be based on the improved classification and identification of Treg cell subsets to further determine the changes in number and function of these cell subsets in AIBD.

### CD8^+^ Treg Cells

While CD4^+^ Treg cells have attracted much attention for their important role in the regulation of immune homeostasis, recent findings have also identified a subset of CD8^+^ T cells with immunoregulatory functions, known as CD8^+^ Treg cells (71–73). Till date, three subsets of CD8^+^ Treg cells have been identified: CD8^+^ CD25^+^ T cells, CD8^+^ CD122^+^ T cells, and CD8^+^ CXCR3^+^ T cells. CD8^+^ CD25^+^ Treg cells express surface CTLA-4 and GITR, as well as intracellular FoxP3, similar to that observed in CD4^+^ Treg cells (74). Meanwhile, CD8^+^CD25^+^ Treg cells could directly contact CD4^+^ CD25^−^ T cells and down-regulate the expression of IL-2Rα, thus suppressing the growth of target cells (74, 75). In addition, human CD8^+^ CXCR3^+^ T cells were found to suppress the expression of IFN-γ in immune cells by secreting IL-10, similar to the observed mechanism in murine CD8^+^ CD122^+^ Treg cells (76–78). CD8^+^ FoxP3^+^ Treg cells were also found to play an important role in the up-regulation of FoxP3 and proliferation of CD4^+^ CD25^+^ Treg cells (79). Although an association between CD8^+^ Treg cells and several autoimmune diseases, such as SLE and experimental autoimmune encephalomyelitis (EAE) has been identified (80, 81), evidence linking CD8^+^ Treg cells to AIBD is still lacking.

## Breg Cells in AIBD

Breg cells were first identified as a subset of IL-10-producing B cells, and were first described by Mizoguchi and Bhan (82). The exact surface markers present in human Breg cells remain unclear. Different B cell subsets in circulation have suppressive regulatory functions that are dependent on IL-10, namely CD19^+^ CD24^high^ CD38^high^ Breg cells, CD19^+^ CD24^high^ CD27^+^ Breg cells, and CD25^high^ CD27^high^ CD86^high^ CD1^high^ Breg cells (83–85). Recently, other Breg subsets, known as TGF-β- expressing Breg cells, have also been identified (86). In autoimmune diseases, such as SLE, multiple sclerosis, lupus nephritis, and type 1 diabetes mellitus, Breg cells are found to be dysfunctional in terms of immune suppression, leading to the breakdown of self-tolerance (87–92). In addition, adoptive transfer of Breg cells ameliorated inflammatory symptoms in animal models of many autoimmune diseases, including SLE, type I diabetes, and contact hypersensitivity (93–96).

### Breg Cells in Pemphigus Vulgaris

**Figure 3:**
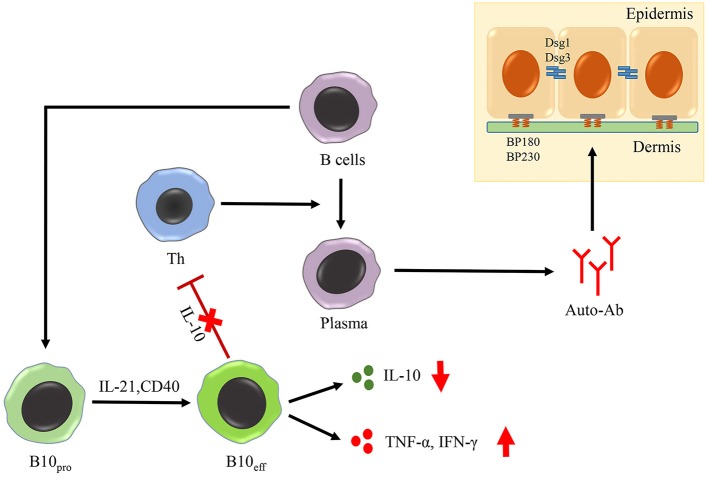
Role of Breg cells in AIBD. The maturation of B10_pro_ cells into B10_eff_ cells requires IL-21- and CD40-dependent cognate interactions with T cells. In PV, B10_eff_ cells is impaired in the functions of secreting IL-10 and down-regulating the production of IFN-γ in CD4^+^ T cells. Moreover, Breg cells from BP patients acquire the function of producing TNF-α and IFN-γ, two important inflammatory cytokines in the pathogenesis of BP.

Activated B cells are generally considered to be pathogenic regulators in patients with PV via the secretion of autoantibodies targeting Dsg3 (97). However, little attention has been paid to the function of Breg cells in the pathogenesis of PV. Several studies have shown that the frequency of CD19^+^ CD24^high^ CD38^high^ Breg cells is significantly increased in the blood of patients with PV compared to that in healthy individuals, both in the active group and in remittent group (98, 99). Breg cells from patients with PV displayed impaired IL-10 expression, even when they were activated over a longer period of time, compared to the healthy Breg cells (98). Therefore, the Breg cells in PV did not directly regulate the humoral response, and rather lost their ability to down-regulate the production of IFN-γ in CD4^+^ T cells (Figure 3). In addition, after being treated with rituximab or intravenous immunoglobulin (IVIG), patients with PV, showing complete remission, had a significantly higher proportion of IL-10-secreting Breg cells and increased levels of IL-10 compared to those who showed no response and incomplete remission (100, 101). Maturation of IL-10-secreting progenitor B (B10_pro_) cells into functional IL-10-secreting effector B (B10_eff_) cells has been confirmed to require IL-21- and CD40-dependent cognate interactions with T cells (102) (Figure 3). Whether patient-derived B10 cells in PV display a defective interaction with T cells, or fail to respond to IL-21 cytokines warrants further investigation.

### Breg Cells in Bullous Pemphigoid

In 2007, Kabuto et al. had found no significant difference in the frequencies of CD19^+^ CD24^high^ CD38^high^ B cells between patients with BP and healthy controls (99). However, in our recent research, we found the frequency of circulating CD19^+^ CD24^high^ CD27^+^ Breg cells and IL-10^+^CD19^+^ Breg cells to be increased in patients with BP. In addition, these patient-derived Breg cells displayed impaired function regarding suppression of CD4^+^ T cell activation and autoantibody production, despite secreting high levels of tumor necrosis factor-α (TNF-α) and IFN-γ (Figure 3). Moreover, the TNF inhibitor etanercept could inhibit autoantibody production *in vitro* (103). According to our results, Breg cells in patients with BP displayed increased production of inflammatory cytokines (mainly TNF-α). Although there is an apparent inconsistency between the pro-inflammatory phenotype and weaker immunosuppressive function of Breg cells in patients with BP, recent studies have proposed immunosuppression not be controlled by a devoted Breg cell lineage with a specific phenotype; rather, it is rather the result of a dynamic balance between multiple B cell subsets and other cells within the immune system (104). Therefore, functional flexibility of the Breg subset may be responsible for the difference in phenotypic vs. functional observations. Further analysis of the Breg phenotype will improve our understanding of its role in BP, and animal models should also be used to study the function of Breg cells in AIBD.

## Other Regulatory Cells

In addition to the cells described above, there are several other cells that have been reported to possess immunomodulatory functions, including DCs, macrophages, MDSCs, and MSCs. Function of DCs is related to their maturation state: immature DCs promote tolerogenic response by expressing anti-inflammatory molecules, such as IL-10 and TGF-β, whereas mature DCs promote immunogenic responses (105, 106). In addition, macrophages can be divided into three populations: classically activated macrophages, wound-healing macrophages, and regulatory macrophages (107). Regulatory macrophages produce high levels of IL-10 and low levels of IL-12, thereby dampening the immune response and limiting inflammation (108, 109). Moreover, MDSCs could also produce a large array of anti-inflammatory molecules, including arginase-1 (ARG1), TGF-β, IL-10, and indoleamine 2,3-dioxygenase (IDO), and have the ability of suppressing T cell response and promoting the functions of Treg cells (110–112). Furthermore, MSCs have been proven to have immunoregulatory effect on innate (NK cells) and adaptive immune system (DCs, B cells, and T cells) by cellular contact and/or secretion of regulatory molecules, such as TGF-β1, HGF, IDO, Prostaglandin E2, and IL-10 (113–116). MSCs also possess the ability to promote the function of Treg cells, including CD4^+^ CD25^bright^ FoxP3^+^ Treg cells and Tr1 cells (117).

Taken together, besides the Treg and Breg cells, many other kinds of cells also have regulatory functions on immune system, and are related to the pathology and/or treatment of autoimmune diseases. However, little is known about the role of these cells in AIBD. One case report had shown CTLA4-overexpressing adipose tissue-derived MSCs transplantation could improve the clinical symptoms of a dog with pemphigus foliaceus (118), suggesting the therapeutic potential of these regulatory cells in AIBD. Further studies are recommended to investigate the roles of these regulatory cells in AIBD, as well as for its treatment.

## Complement Regulatory Proteins in AIBD

CRPs refer to an important class of regulatory proteins in the complement system that controls enzymatic cascades, assembly of the membrane attack complex, and homeostasis of the complement system (119, 120); they include CD55 (decay accelerating factor), CD59 (membrane inhibitor of reactive lysis; MIRL), CD35 (type 1 complement receptor; CR1), and CD46 (membrane cofactor protein; MCP). Since the over-activation of the complement system could injure the host, effectors of complement activation need to be tightly regulated by complement regulatory proteins to maintain the balance between efficient destruction of harmful factors and unwanted over-activation of host tissue (121). Dysregulation of these proteins is related to many autoimmune diseases, including SLE, autoimmune hemocytopenias, and rheumatoid arthritis (122–126).

**Figure 4:**
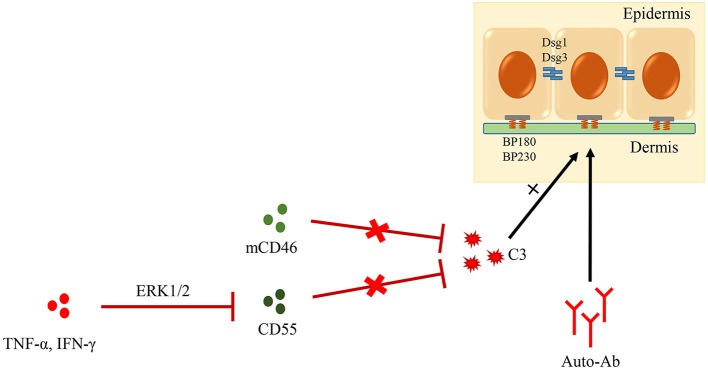
Role of CRPs in AIBD. Complement activation is one of the pivotal steps in BP development. mCD46 and CD55, two important CRPs, could inhibit autoantibody-mediated complement activation. Additionally, CD55 is down-regulated by TNF-α and IFN-γ though ERK1/2 signaling pathway in BP lesions.

Although DIF studies of patients with PV often show C3 deposits at the keratinocyte surface, blister formation in PV has been proven to be independent of complement (127). Complement activation via the classical pathway is one of the pivotal steps in BP development (27). However, little is known about the role and mechanism of CRPs in BP. In our latest study, we examined the role of CD46 and CD55 in the pathogenesis of BP; results showed that sCD46 was up-regulated in both serum and blister fluid of the patients, and that its expression was positively correlated with the levels of anti-BP180 antibody and C3a. Importantly, the levels of mCD46 and CD55 were decreased in skin lesions of patients with BP. While the depletion of CD46 or CD55 enhanced C3 deposition and autoantibody-mediated complement activation, exogenous CD46 or CD55 significantly inhibited this phenomenon (128, 129) (Figure 4). These data suggested CD46 and CD55 to be key inhibitors of complement activation and that their down-regulation is involved in the pathogenesis of BP.

## Conclusion

According to the previous results and our recent investigations, it is clear that the components of immunoregulatory system, including Treg cells, Breg cells, and CRPs, are important for the development of autoimmune disorders in AIBD. Recently, pharmacological approaches targeting Treg cells to restore their impaired function have been considered viable therapeutic methods for some autoimmune diseases (130–133), clarifying the importance of immunoregulatory system in the pathogenesis of these diseases, and showing the latent capacity of regulatory immune cells and molecules in these diseases. However, present studies have relied on the identification of changes in cell counts and functions, without exploring its role in disease development and treatment of AIBD in depth. Since the animal models of AIBD have been well-developed, more studies based on animal models would be required to expound the role and possible therapeutic applications of these cells and molecules in AIBD. Studies exploiting their suppressive characteristics should also be carefully designed to obtain maximum clinical benefits.

## Ethics Statement

All the patients gave written informed consent for the publication of the pictures.

## Author Contributions

TC and GW conceived the idea. TC, SS, HF, and BL contributed to the writing of the manuscript. TC contributed to figure preparation for the manuscript.

### Conflict of Interest Statement

The authors declare that the research was conducted in the absence of any commercial or financial relationships that could be construed as a potential conflict of interest.

## Abbreviations

AIBD: autoimmune bullous dermatoses
ARG1: arginase-1
BP: bullous pemphigoid
Breg cells: regulatory B cells
B10eff cells: functional IL-10-secreting effector B cells
B10pro cells: IL-10-secreting progenitor B cells
CRPs: complement regulatory proteins
CTLA-4: cytotoxic T-lymphocyte associated protein 4
DCs: dendritic cells
DIF: direct immunofluorescence
Dsg: desmoglein
EAE: experimental autoimmune encephalomyelitis
H&E: haematoxylin and eosin
IDO: indoleamine 2,3-dioxygenase
IIF: indirect immunofluorescence
IL: interleukin
IVIG: intravenous immunoglobulin
mAb: monoclonal antibody
BMZ: basement membrane zone
MCs: mast cells
MDSCs: myeloid-derived suppressor cells
MSCs: mesenchymal stromal cells
NC16A: non-collagenous 16A
NK cells: natural killer cells
nTreg cells: natural Treg cells
PV: pemphigus vulgaris
SLE: systemic lupus erythematosus
TGF-β: transforming growth factor-β
TNF-α: tumor necrosis factor-α
Treg cells: regulatory T cells
Tr1 cells: regulatory type 1 cells.
