# Supplementary material for: Role of Regulatory Immune Cells and Molecules in Autoimmune Bullous Dermatoses
Source: Front Immunol. 2019 Aug 2;10:1746. doi: 10.3389/fimmu.2019.01746 (PMC6688483; doi:10.3389/fimmu.2019.01746)
Supplement: Supplementary file 1 [file Image_1.pdf]

Supplementary Material

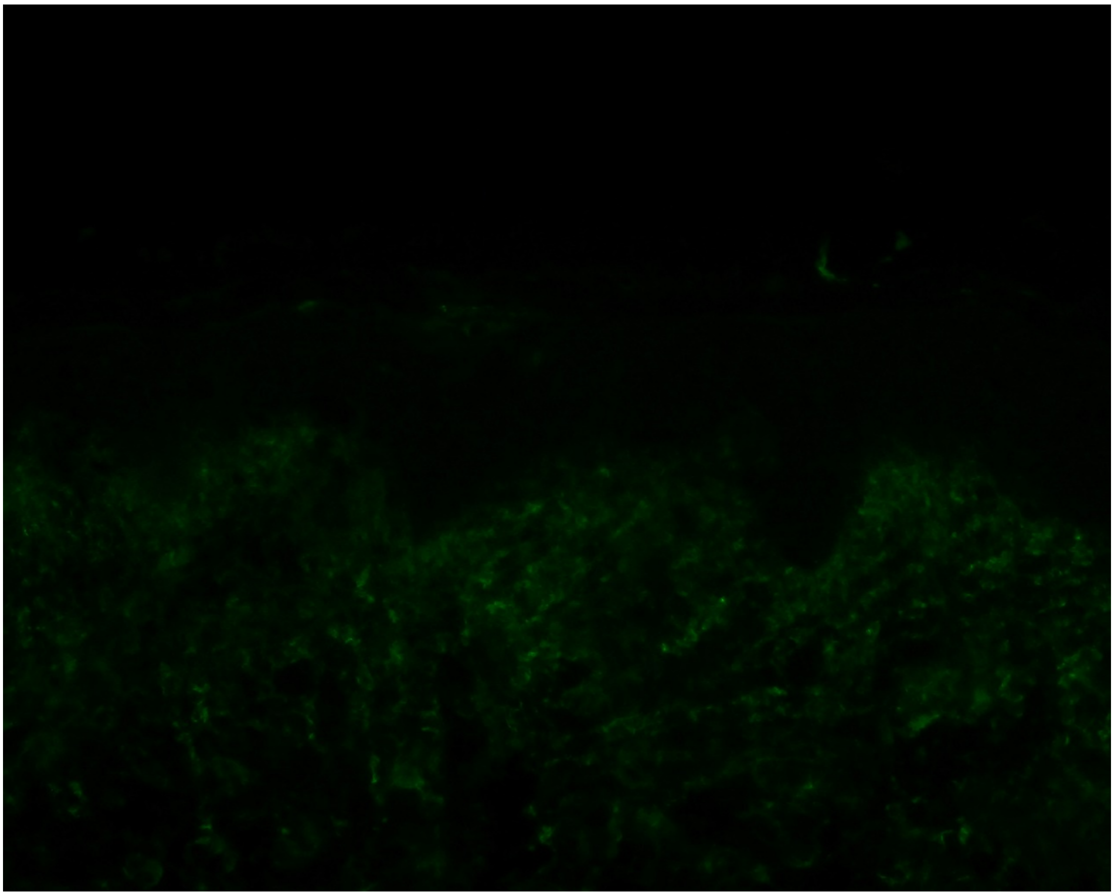

Supplementary Figure 1. IgG staining for normal skin. DIF of normal skin from healthy donor shows no specific fluorescence in the epidermis or the dermoepidermal junction.
